# Demographic and clinical characteristics of free-text writers in chronic pain patient intake questionnaires

**DOI:** 10.1080/24740527.2021.2016031

**Published:** 2022-02-18

**Authors:** Rachel Roy, Jordana L. Sommer, Ryan Amadeo, Kristin Reynolds, Kayla Kilborn, Brigitte Sabourin, Renée El-Gabalawy

**Affiliations:** a Department of Anesthesiology, Perioperative and Pain Medicine, Max Rady College of Medicine, Rady Faculty of Health Sciences, University of Manitoba, Winnipeg, Manitoba, Canada; b Department of Psychology, Faculty of Arts, University of Manitoba, Winnipeg, Manitoba, Canada; c Department of Psychiatry, Max Rady College of Medicine, Rady Faculty of Health Sciences, University of Manitoba, Winnipeg, Manitoba, Canada; d Department of Clinical Health Psychology, Max Rady College of Medicine, Rady Faculty of Health Sciences, University of Manitoba, Winnipeg, Manitoba, Canada; e CancerCare Manitoba, Winnipeg, Manitoba, Canada

**Keywords:** chronic pain, free-text, pain facility, health, health care use, patient communication

## Abstract

**Background:**

Chronic pain is a prevalent and burdensome problem within the Canadian health care system, where the gold standard treatment occurs at multidisciplinary pain facilities. Patient intake questionnaires (PIQs) are standard practice for obtaining health information, with many patients including free-text (e.g., writing in margins of questionnaires) on their PIQs.

**Aims:**

This study aims to quantitatively examine whether and how patients who include free-text on PIQs differ from those who do not.

**Methods:**

We retrospectively analyzed 367 PIQs at a Canadian pain facility in Winnipeg, Canada. Patients were categorized into free-text (i.e., any text response not required in responding to questions) or no free-text groups. Groups were compared on sociodemographics, pain, health care utilization, and depressive symptoms with independent samples *t*-tests and chi-square analyses.

**Results:**

Patients with free-text compared to those without had more sources of pain (6.66 vs. 4.63), longer duration of pain (123.2 months vs. 68.1 months), and a greater proportion of past pain conditions (66.3% vs. 55.2%). Additionally, they had tried more treatments for their pain, had seen more specialists, had tried more past medications, were currently on more medications, and had undergone more tests. No differences were identified for depressive symptoms across groups.

**Conclusions:**

This study is the first to examine patient and health-related correlates of free-text on PIQs at a Canadian pain facility. Results indicate that there are significant differences between groups on pain and health care utilization. Thus, patients using free-text may require additional supports and targeted interventions to improve patient–physician communication and patient outcomes.

## RÉSUMÉ

**Contexte**: La douleur chronique est un problème répandu et pénible dans le système des soins de santé au Canada, où le traitement de référence se produit dans un centre multidisciplinaire de la douleur. Les formulaires d‘admission du patient sont une pratique courante pour obtenir des renseignements sur la santé, et de nombreux patients y incluent du texte libre (en écrivant, par exemple, dans les marges du formulaire).

**Objectifs**: Cette étude vise àexaminer quantitativement si et comment les patients qui incluent du texte libre sur leur formulaire d‘admission diffèrent de ceux qui ne le font pas.

**Méthodes**: Nous avons analysé rétrospectivement 367 formulaires d‘admission dans un centre canadien de traitement de la douleur à Winnipeg, au Canada. Les patients ont été classés en deux groupes : avec texte libre (c‘est-à-dire ayant inscrit une réponse sous forme de texte non requis au moment de répondre aux questions) ou sans texte libre. Les groupes ont été comparés en ce qui concerne les critères sociodémographiques, la douleur, l‘utilisation des soins de santé et les symptômes dépressifs, à l‘aide de tests t et chi carré pour échantillons indépendants.

**Résultats**: Les patients avec texte libre par rapport à ceux sans texte libre avaient plus de sources de douleur (6,66 comparativement à 4,63), une plus longue durée de la douleur (123,2 mois comparativement à 68,1 mois) et une plus grande proportion de douleur par le passé (66,3 % comparativement à 55,2 %). De plus, ils avaient essayé d‘autres traitements pour leur douleur, avaient vu plus de spécialistes, avaient essayé plus de médicaments par le passé, prenaient à ce moment plus de médicaments et avaient subi plus de tests. Aucune différence n‘a été recensée pour les symptômes dépressifs entre les groupes.

**Conclusions**: Cette étude est la première à examiner les corrélats liés au patient et à la santé de l‘existence de texte libre sur les formulaires d‘admission dans un centre de traitement de la douleur au Canada. Les résultats indiquent qu‘il existe des différences importantes entre les groupes en ce qui concerne la douleur et l‘utilisation des soins de santé. Ainsi, les patients utilisant le texte libre peuvent avoir besoin de soutien additionnel et d‘interventions ciblées pour améliorer la communication patient-médecin et les résultats du patient.

Chronic pain is a pervasive, burdensome problem for the Canadian health care system, patients, and physicians and is defined as pain that persists for over 3 months.^1^^,2^ Prevalence estimates of chronic pain in the Canadian population are typically estimated at one in five, and rates are further elevated among older adults and women.^3,4^ The cost associated with chronic pain is estimated at CA$37 billion per year,^5^ with some estimates as high as $56 to $60 billion per year.^2^ Pain conditions are often multifaceted and complex, impacting patients’ physical and mental health. Increased risk of comorbid mental health conditions, feelings of hopelessness, low quality of life, and suicidality can be seen in individuals experiencing chronic pain.^1,4,6–9^ The recommended standard for treatment of chronic pain is a multidisciplinary approach provided by community or hospital-based specialty chronic pain treatment facilities.^10^

## Waiting Lists

Due to the aforementioned high chronic pain prevalence rates, Canadian pain treatment facilities are often burdened by long waiting lists,^11–14^ with many individuals experiencing deteriorating health-related quality of life and overall mental health while waiting.^15^ A study of patients on waiting lists across Canada revealed that depression, anxiety, and distress levels increased after only a 3-month wait time.^16^ This is a concern because these patients are already at high risk for mental health comorbidities,^1,3,17^ and anxiety and depressive symptoms can exacerbate the severity of chronic pain and associated disability.^3,18^ Additionally, patients who attend specialty pain treatment facilities often have more complex pain conditions and are more likely to be unemployed and have mental health comorbidities than those who remain in primary care.^19^ This highlights the fact that patients presenting at these facilities have complex pain presentations including comorbid mental health problems, and when faced with long wait times, these negative effects may be heightened.

## Patient–Provider Communication

While on long waiting lists for treatment, patients may receive little or no communication from their pain physicians until they have their first appointment. Limited communication between patients and their health care providers has been found to have a variety of negative effects on emotional and physical outcomes (e.g., social function and vitality).^20,21^ Additionally, pain patients have identified feelings of frustration and hopelessness with the health care system.^22–24^ They have also reported feeling a lack of trust and respect from health care providers, who they perceive as dismissing their symptoms and/or suspecting them of drug-seeking behaviors.^24^ Proper patient–physician communication (e.g., involvement of patient in decision making; accessibility of physician between visits) has been linked to better outcomes, including increased patient recall, adherence, and satisfaction,^21^ and is important for improved pain management.^25–27^ Improving communication may also reduce overall frustration with the health care system.^22^

One way in which communication first takes place between patient and provider is through the use of patient intake questionnaires (PIQs). The purpose of PIQs is to gather relevant health and psychosocial information prior to a patient’s first appointment. They are regularly used by Canadian treatment teams to inform clinical decision making.^28–31^ Previous research has looked at characteristics of patients on the waiting list for multidisciplinary pain treatment facilities^16,19^; however, this research has not identified characteristics of patients who include notable amounts of free-text on their PIQs. Across facilities, free-text in paper-based questionnaires outside of standardized pain and psychosocial health questions is the norm. Although the clinical relevance of this text and whether there are differences between those who include free-text from those who do not remain unknown, it has been previously suggested that free-text may be a form of communication worth examining.^32^

This exploratory study aims to quantitatively examine patient profiles (i.e., sociodemographics, pain characteristics, health care utilization, and depressive symptoms) of those who include free-text (i.e., text that is not part of a question) in PIQs compared to those who do not (of note, a second study by our group is qualitatively examining these free-text entries). To date, we are not aware of previous research in this area. We hypothesize that free-text writers will have more complex pain defined by pain characteristics, health care utilization, and relevant psychological features (higher depressive symptoms). This investigation may be particularly timely given the transition of many PIQs to electronic format, where important clinical indicators and additional forms of communication may be lost. Having a better way of identifying who may feel unheard by the health care system may lead to enhanced communication, thus improving patient outcomes.

## Method

### Sample

We retrospectively reviewed a convenience sample of PIQs of 367 patients at the Health Sciences Center Pain Management Center in Winnipeg, Manitoba, Canada. Data were collected between January 20, 2015, and February 12, 2018. A total of 396 patients were screened; however, only 367 were identified as having completed the full-length questionnaire and thus were included in this study. Patient lists were obtained in paper form from the pain clinic of recent appointments from the past week, and patient questionnaires were searched and collected via secure hospital database. Patients were referred to the Pain Management Center by their general practitioner or another medical specialist. Patients are referred to the pain clinic to receive more specialized services (that may not be provided by primary care); however, services are not discontinued by their primary care provider. We categorized patients into either free-text or no free-text groups. Free-text was defined as any text response that was not required by the stated question (e.g., free-text in margins, additions to fixed choices, comments about questionnaire). Use of these data for secondary analysis was approved by the University of Manitoba Research Ethics Board (#HS22865(H2019:203)). We obtained approval from the director of the Pain Clinic to access these data and patient consent was not required by the research ethics board due to the retrospective nature of the study.

### Measures

Patients with chronic pain on the Health Sciences Center Pain Management Center’s waiting list were mailed a 20-page PIQ by administrative staff. The patients were required to complete and return these questionnaires prior to receiving an initial appointment at the clinic. The questionnaire included multiple categories: patient demographic data, pain history, treatment history, review of systems, family history, occupational history, social history, pain and sleep, appetite, and finances. In addition, several previously validated and reliable scales were included: the Pain Catastrophizing Scale (PCS),^33^ Patient Health Questionnaire–9 (PHQ-9), and Brief Pain Inventory–Pain Interference Scale.^34^ For the purpose of this study, we looked only at select variables to understand patient characteristics in relation to free-text. These variables fell into four main categories: sociodemographics, pain characteristics, health care utilization, and mental health characteristics.

#### Sociodemographics

We included sociodemographic characteristics (i.e., age, sex, marital status, education history, employment status, country of origin). Age was assessed continuously and sex (male or female), marital status (married/common law or widowed/separated/divorced or single/never married), education history (high school/less than high school or some college/higher education), employment status (yes or no), and country of origin (Canada or other) were assessed as categorical variables.

#### Pain Characteristics

Questions assessing pain characteristics included, “How long have you had your painful condition?” specified in months, “Have you had other painful conditions in the past?” (yes, no), and “Where is your pain located?” (head, arm, abdomen, mid-back, etc.), which was recoded as a continuous variable to capture the number of sources of pain. To capture information on pain severity, patients were asked “What is the usual level of pain you had during the last week?” (0 = *no pain* to 10 = *worst pain imaginable*; assessed continuously). Patients also completed The Brief Pain Inventory–Pain Interference Scale^34^ and the PCS. The Brief Pain Inventory–Pain Interference Scale measures how significantly patients feel their pain has interfered with their life across a number of domains, including general activity, mood, walking ability, normal work, relations with others, sleep, and life enjoyment using a sliding scale (0 = *does not interfere*, 10 = *completely interferes*). Total scale scores were computed to create a continuous score of pain interference, with higher scores indicating higher pain interference. The PCS measures patient pain catastrophizing using 13 items on a 5-point scale. Patients rate statements such as, “When I am in pain … ” (e.g., I worry all the time about whether the pain will end, I feel I can’t go on), with responses ranging from 0 (*not at all*) to 4 (*all the time*). A total score is computed by summing the 13 items and ranges from 0 to 52, with higher scores indicating greater pain catastrophizing. The PCS has demonstrated reliability and validity in measuring patient pain catastrophizing.^33^

#### Health Care Utilization

Patients self-reported their medical history, including listing all pain medications they were currently using and the medications they had tried for their pain in the past. Two continuous variables were created, including total number of pain medications currently used and total number of pain medications tried in the past. Additionally, patients were asked, “Since your pain began, which of the following people have you seen about it?” (e.g., acupuncturist, psychologist, occupational therapist) to assess the number of specialists they have seen regarding their pain. Related, they were asked about treatment history: “Have you tried any of the following for your pain?” (e.g., nerve blocks, heat therapy, biofeedback) to assess the number of alternative treatments tried. To assess number of medical investigations, patients were asked “Which tests/treatments have been done?” (e.g., X-ray, magnetic resonance imaging). These questions were used to compute continuous variables representing the number of specialists seen, number of treatments tried, and number of tests undergone, respectively. Additionally, a categorical variable was created based on alternative treatments (yes, no) to capture whether patients had ever tried any of these (e.g., biofeedback, nerve blockers, acupuncture, heat therapy, hypnosis, manipulation, ultrasound, massage, psychotherapy, exercise, bedrest, traction). Patients were also asked how many hospital and emergency visits they had regarding their pain. The hospitalization and emergency visit variables were categorized as “Have you ever visited the emergency room for your pain?” (yes, no) and “Have you ever been hospitalized for your pain?” (yes, no). These variables relate to lifetime pain-related visits. Additionally, to capture accident-related pain, two questions asking whether the visit was related to either a motor vehicle accident or a work accident were collapsed into a single accident-related variable measuring whether the visit was related to a workplace or motor vehicle accident (yes, no).

#### Depressive Symptoms

The PHQ-9 has been validated for assessing depressive symptoms.^35^ Patients are asked, “Over the last two weeks, how often have you been bothered by any of the following problems?” (e.g., little interest or pleasure in doing things, feeling down, depressed, or hopeless). They respond with one of four choices: *not at all* (scored as 0), *several days, more than half the days, nearly every day* (scored as 3). This questionnaire produces a continuous depressive score ranging from 0 to 27, with higher numbers indicative of greater depressive symptoms. In addition, we analyzed those who met the cutoff of 15 for clinically significant depression and depressive symptom severity categories (no/minimal symptoms, mild symptoms, moderate symptoms, severe symptoms).^35^

### Analytic Strategy

We first assessed the prevalence of free-text use among the PIQs. We then used independent samples *t*-tests and cross-tabulations with chi-square analyses to demonstrate differences in sociodemographics, pain characteristics, health care utilization, and depressive symptoms between the free-text group versus those in the no free-text group. To examine whether there were differences in pain locations according to free-text writing, we first ran a two-step cluster analysis to identify underlying pain location clusters/subgroups within the sample, with the Bayesian information criterion to assess model fit. A chi-square analysis then assessed whether there were differences in the prevalence of each cluster among those who did and did not include free-text.

## Results

Out of the 396 participants screened, 295 (80.4%) had free-text on their PIQs. Among those who used free-text, the average age was 54.12 years old, and the majority were female (61.0%). No sociodemographic factors significantly differed across groups (see Table 1). We used single imputation for validated self-report scales with more than 5% missing data. Prior to imputation, among those who completed at least some of the PCS, PHQ-9, and Pain Interference Scale, there were 19.7%, 11.0%, and 14.2% with incomplete responses, respectively. Sensitivity analyses using the nonimputed variables showed results did not differ across the PHQ-9 and the PCS. However, results differed comparing the imputed and nonimputed data for pain interference; when imputation was not done, free-text writers had more pain interference compared to those with no free-text. Additionally, we investigated whether the amount of missing data varied across free-text and no free-text groups using a missing value analysis among each group. This analysis showed groups did not differ in the amount of missing data, with 8.5% of values missing among those without free-text and 8.6% of values missing among those with free-text.

**Table 1.** Sample characteristics of those with free-text vs. those without

**Sociodemographics**	Free-text	No free-text	Chi-square/*t*-statistic
Sample size	295 (80.4%)	72 (19.6%)	
Education			1.27
High school or less	152 (53.3%)	42 (60.9%)	
Some college or higher	133 (46.7%)	27 (39.1%)	
Currently employed	106 (36.3%)	29 (40.3%)	0.39
Marital status			0.54
Married or common law	45 (60.3%)	173 (63.4%)	
Widowed, separated, or divorced	64 (22.3%)	13 (18.3%)	
Single/never married	50 (17.4%)	13 (18.3%)	
Sex			2.90
Male	115 (39.0%)	36 (50.0%)	
Female	180 (61.0%)	36 (50.0%)	
Age^a^	54.12 (14.98)	56.63 (14.20)	1.23
Canadian born^b^	266 (91.4%)	61 (84.7%)	0.09
**Pain characteristics**			
Number of sources of pain^a^	6.66 (4.64)	4.63 (3.23)	−4.35***
Pain location cluster			8.89**
Widespread pain cluster	124 (42.8%)	17 (23.6%)	
Localized pain cluster	166 (57.2%)	55 (76.4%)	
Duration of pain condition (months)^a^	123.24 (129.36)	68.09 (72.63)	−4.51***
Usual past week pain^a^	6.67 (2.01)	6.77 (1.68)	0.417
Had other painful conditions in the past	169 (66.3%)	34 (52.3%)	4.36*
Accident-related pain	64 (22.5%)	14 (19.7%)	0.61
Pain interference (past 24 h)^a^	50.42 (17.81)	45.91 (18.10)	−1.83
Pain Catastrophizing Scale^a^	27.35 (13.30)	27.95 (13.15)	0.32
**Health care utilization**			
Gone to emergency room for pain condition^c^	144 (49.5%)	28 (38.9%)	2.60
Been hospitalized for pain condition^c^	48 (16.7%)	7 (9.7%)	2.18
Number of alternative treatments tried^a^	4.53 (3.22)	3.68 (3.19)	−2.02*
Have accessed any type of alternative treatment	270 (92.5%)	61 (85.9%)	3.05
Number of tests undergone (e.g., X-ray)^a^	2.24 (1.30)	1.74 (1.16)	−2.92*
Number of specialists seen^a^	4.32 (2.39)	3.20 (2.09)	−3.89***
Number of past medications tried^a^	3.16 (2.39)	2.05 (0.95)	−4.81***
Number of current medications^a^	6.46 (3.68)	5.00 (3.73)	−2.81*
**Mental health**			
Patient Health Questionnaire-9 (continuous depressive score)^a^	12.42 (6.86)	11.89 (7.04)	−0.57
Meets cutoff for clinically significant depression (15 or higher)^a^	106 (37.2%)	23 (34.3%)	0.19
Severity of depressive scores			0.55
No/minimal symptoms	42 (14.8%)	12 (17.9%)	
Mild symptoms	67 (23.7%)	15 (22.4%)	
Moderate symptoms	68 (24%)	17 (25.4%)	
Severe symptoms	106 (37.5%)	23 (34.3%)	

Numbers represent descriptives among those who used free-text (cross-tabulations).

^a^Values represents *M* (SD) and *t*-statistic.

^b^Due to small cell sizes, participants’ country of origin was categorized into Canadian born or other.

^c^Variable captures pain-related lifetime without a specific time frame for hospital or emergency visits (i.e., “Have you ever been to the emergency room for your pain?” and “Have you ever been hospitalized for your pain?”).

*P < 0.05, **P < 0.01, ***P < 0.001.

### Pain Characteristics

Those who had instances of free-text on the PIQ were found to be significantly different in terms of the number of sources of their pain, duration of their pain condition in months, and whether or not they had other past pain conditions. Individuals in the free-text group on average reported 6.66 sources of pain versus 4.63 sources in the no free-text group (*t* = −4.35, *P* < 0.001). Duration of pain condition was almost double in the free-text group (123.24 months) compared to the no free-text group (68.09 months; *t* = −4.51, *P* < 0.001). Furthermore, individuals in the free-text group were significantly more likely to have had other painful conditions in the past (66.3%) than those in the no free-text group (52.3%; χ^2^ = 436, *P* < 0.05). There was no significant difference found between free-text writers and those with no free-text in terms of usual pain experienced in the last week (i.e., pain severity). Additionally, no significant differences were found on the PCS in terms of pain catastrophizing across groups. The two-step cluster analysis on pain location revealed two clusters. The first cluster, “widespread pain,” was characterized by a high prevalence of multiple areas of pain (e.g., 87.1% with neck pain, 50.8% with head pain). The second, “localized pain,” was characterized by a high prevalence endorsing one area responsible for their pain (majority lower back; 71.4%). Widespread pain was more common among those with free-text (42.8%) compared to those without free-text (23.6%), and localized pain was prevalent in just over half (57.2%) of those with free-text and among nearly three-quarters (76.4%) of those without free-text.

#### Health Care Utilization

Participants who had instances of free-text were found to have, on average, significantly greater health care utilization in terms of treatments tried, tests undergone, specialists seen, and number of both past and current medications. The free-text group on average tried 4.53 alternative treatments, versus 3.68 in the no free-text group (*t* = −2.02, *P* < 0.05). Additionally, they underwent more tests on average (2.24) than the no free-text group (1.74; *t* = −2.92, *P* < 0.05). Free-texters also saw more specialists (4.32) than non-free-texters (3.20; *t* = −3.89, *P* < 0.001), had tried more medications in the past (3.16 vs. 2.05; *t* = −4.81, *P* < 0.001), and were currently on more medications (6.46 vs. 5.00; *t* = −2.81, *P* < 0.05).

#### Depressive Symptoms

No significant differences were found between free-text writers versus those with no free-text in terms of depressive symptomatology (i.e., PHQ-9).

## Discussion

To the best of our knowledge, this study is the first to examine differences between individuals adding free-text and those not adding any free-text on a comprehensive PIQ at a Canadian multidisciplinary specialty pain facility. Results revealed that free-text writers are demographically similar and have comparable levels of depressive features, pain catastrophizing, and current pain severity. However, they significantly differ in other pain indicators (i.e., greater number of sources of pain, pain comorbidities, and duration) and have higher health care utilization compared to those with no free-text. In addition, free-text writers’ pain profiles appear unique, characterized by more widespread pain, compared to more localized pain in those with no free-text. This suggests that free-text writers may have a more substantive help-seeking history related to greater complexity of chronic pain.

### Pain Characteristics

A significant difference was found between free-text writers versus those with no free-text on various pain-related variables indicative of a more complex pain presentation. Specifically, free-text writers had significantly more sources of pain than those with no free-text, the duration of their current pain condition was longer, and they were more likely to have had other pain conditions in the past and widespread pain compared to localized pain. All of these variables suggest the possibility that pain complexity and duration of overall pain experience may influence whether or not people write free-text on their PIQs. Related, in a separate manuscript (being prepared for submission) including a qualitative analysis of these PIQs by our group, 32.6% of patients wrote free-text pertaining to information about the duration of their pain.^36^ Given that these individuals have been experiencing pain for longer, they may have more to say about their pain journey and/or experiences that were not adequately captured by this PIQ. In line with the health care use findings, discussed in the Health Care Utilization section below, this longer duration likely translates to greater experience with various pain treatments. Interestingly, current pain severity was not found to be higher in the free-text group than in the no free-text group and pain interference was only just approaching significance, at an average score of 50.42 for free-text writers and 45.91 for those with no free-text. This may support the hypothesis that pain duration and time spent interacting with the health care system may be influencing free-text rather than the pain experience itself (i.e., severity of pain, catastrophizing, interference). Therefore, duration of pain condition and multiple sources of pain in those who write free-text may demonstrate that these patients have unique pain-related experiences compared to those without free-text. Surprisingly, no significant differences were found among free-text writers versus those with no free-text in terms of pain catastrophizing.

### Health Care Utilization

Significant differences in health care utilization were identified between free-text writers and those with no free-text. Particularly, those utilizing free-text on their PIQs had tried significantly more treatments than those with no free-text. Furthermore, they had seen more specialists, had undergone more tests, and had tried more medications in the past. They were also currently taking a significantly higher number of medications than those in the no free-text group. Given the more intensive health care history, it is possible that patients believe that the standard PIQs do not sufficiently capture their help-seeking history, resulting in the use of free-text. Previous research has shown that this free-text on questionnaires may be a form of communication.^32^ It is also possible that these patients may be experiencing feelings of frustration or helplessness,^37^ particularly, frustration stemming around the limitations of health care in terms of their pain management and perceived failure of the health care system to find an effective treatment of their pain. This may result in more free-text writing in attempts to communicate their suffering and feelings of frustration or helplessness with the system. In peripheral support, qualitative research has noted that frustration with the persistent issue of chronic pain is a common feeling for patients.^22,38^ Further, in a sample of veteran patients with pain, patients expressed frustration with lack of continuing care and navigating logistical barriers associated with the health care system.^22^ However, as discussed below, depressive symptoms, which may capture feelings of helplessness, were not significantly different between groups. It is also possible that certain untested patient characteristics are associated with both an increased likelihood to write free-text and to be less responsive to previous pain treatments. Potential characteristics include treatment expectations, baseline self-efficacy, and optimism, which have been found to have an effect on treatment outcomes.^39^ Unfortunately, none of these patient characteristics are captured on the Pain Management Center’s PIQs.

### Depressive Symptoms

Surprisingly, and in opposition to what was hypothesized, there were no significant differences between free-text writers and those with no free-text in terms of depressive symptoms. This is particularly surprising because the pain characteristics associated with free-text (e.g., duration of pain, number of sources of pain, past pain conditions) all relate to pain severity, which has been linked to depression in this population.^18^ However, pain severity was also not significant in this study. These null findings may relate in part to the sample of patients who present at these specialized pain clinics. Patients waiting for service at the pain clinic experience some of the most complex pain, because they often seek out the clinic after having tried numerous other medical treatments, and they also experience high levels of depression as an overall group.^15,18^ For example, moderate or severe depressive symptoms were reported by over half the patients in both groups (see Table 1). This may mean that both free-text writers and those with no free-text are experiencing similar mental health struggles due to the complexity of their pain.

### Limitations

Despite significant findings related to differences between free-text writers and those with no free-text on pain, medical history, and health care utilization–related variables, it is important to note this study’s limitations. The data captured for the purposes of this research are from a single specialty chronic pain treatment facility within Manitoba, and further research is needed to examine whether similar results emerge in facilities in other jurisdictions. This may lead to more information regarding trends among patients on the waiting list and those adding free-text on their questionnaires. Related, we did not analyze common themes across the specific instances of free-text, which may provide more context regarding what free-text may be indicative of or what the free-text writers may be trying to communicate. This research group has prepared a separate manuscript that qualitatively analyzes the free-text content.^36^ Additionally, the data for this study were cross-sectional and self-reported instead of physician assessed. However, patient-reported outcomes are important to gain the patient perspective within pain-related research because pain is often subjective and can be difficult to measure. In fact, patients’ feedback about their experiences has been identified as one of the top 10 priorities to improve patient care in anesthesia.^40^ Additionally, patient engagement has been identified as key to improving patient-centered care, and an important aspect of this has been found to be the incorporation of patient-reported outcomes in their care.^41^ It is also important to note that this is a retrospective analysis of PIQs that may not include all relevant information related to patients (e.g., there was no specific pain diagnosis or race/ethnicity). Finally, due to our use of a convenience sample, results may have limited generalizability.

### Clinical Implications

Although needing further research, our results have important clinical implications regarding patients with chronic pain. Primarily, 80.4% of our sample had free-text on their PIQs, indicating that space for patients to elaborate should be standard practice on questionnaires. This could enhance patient–provider communication and help patients feel heard by their health care providers, which may in turn improve patient outcomes (patient satisfaction, adherence, and pain management).^21,25–27^ Space for free-text responses allows more complex patients to communicate their story from the first point of communication, facilitating improved patient–provider relationships early on. Furthermore, the presence of free-text on PIQs appears to reflect a more complex pain journey and health history. This suggests that free-text writers might require additional supports and better communication with their health care professionals or have unmet treatment needs. Specifically, patients who have more instances of trying to access the health care system (e.g., undergone more tests, tried more treatments, on more medications) and who have more complex pain (e.g., more sources of pain, longer duration with pain condition, and multiple pain conditions in the past) may require targeted interventions to improve both patient–physician communication and patient outcomes. Future research should aim to understand other factors associated with free-text, including health care expenditures and treatment resistance. It is also important to consider differences between groups in light of the evolving transition to electronic PIQs and potential benefits but also downfalls to this approach, including the inability for patients to communicate additional clinical information.
